# Characterization of the Rel_Bbu_ Regulon in *Borrelia burgdorferi* Reveals Modulation of Glycerol Metabolism by (p)ppGpp

**DOI:** 10.1371/journal.pone.0118063

**Published:** 2015-02-17

**Authors:** Julia V. Bugrysheva, Christopher J. Pappas, Darya A. Terekhova, Radha Iyer, Henry P. Godfrey, Ira Schwartz, Felipe C. Cabello

**Affiliations:** 1 Department of Microbiology and Immunology, New York Medical College, Valhalla, New York, 10595, United States of America; 2 Department of Pathology, New York Medical College, Valhalla, New York, 10595, United States of America; Johns Hopkins University School of Medicine, UNITED STATES

## Abstract

The bacterial stringent response is triggered by deficiencies of available nutrients and other environmental stresses. It is mediated by 5'-triphosphate-guanosine-3'-diphosphate and 5'-diphosphate-guanosine-3'-diphosphate (collectively (p)ppGpp) and generates global changes in gene expression and metabolism that enable bacteria to adapt to and survive these challenges. *Borrelia burgdorferi* encounters multiple stressors in its cycling between ticks and mammals that could trigger the stringent response. We have previously shown that the *B. burgdorferi* stringent response is mediated by a single enzyme, Rel_Bbu_, with both (p)ppGpp synthase and hydrolase activities, and that a *B. burgdorferi* 297 rel_Bbu_ null deletion mutant was defective in adapting to stationary phase, incapable of down-regulating synthesis of rRNA and could not infect mice. We have now used this deletion mutant and microarray analysis to identify genes comprising the *rel* regulon in *B. burgdorferi* cultured at 34°C, and found that transcription of genes involved in glycerol metabolism is induced by *rel*_Bbu_. Culture of the wild type parental strain, the *rel*_Bbu_ deletion mutant and its complemented derivative at 34°C and 25°C in media containing glucose or glycerol as principal carbon sources revealed a growth defect in the mutant, most evident at the lower temperature. Transcriptional analysis of the *glp* operon for glycerol uptake and metabolism in these three strains confirmed that *rel*_Bbu_ was necessary and sufficient to increase transcription of this operon in the presence of glycerol at both temperatures. These results confirm and extend previous findings regarding the stringent response in *B. burgdorferi*. They also demonstrate that the stringent response regulates glycerol metabolism in this organism and is likely crucial for its optimal growth in ticks.

## Introduction

Nutritional exigencies and other environmental challenges are met by bacteria through the use of global regulatory pathways [1–4]. The comprehensive shifts in gene expression induced by these regulatory pathways at the transcriptional and post-transcriptional level permit the rapid and dramatic modulation of bacterial growth and metabolism needed to adapt to these challenges. One of these global regulatory responses, the stringent response, is conserved in virtually all bacteria. It was originally described in *Escherichia coli* in association with amino acid scarcity, but has subsequently been shown to be triggered by many other environmental stressors including insufficiencies of iron, glucose and fatty acids [1–4]. It is mediated by the nucleotide alarmones guanosine-3'-diphosphate-5'-diphosphate and guanosine-3'-diphosphate-5'-triphosphate (collectively (p)ppGpp), each with a similar but distinct regulatory potential [1,2,5].

In *E*. *coli*, cytosolic levels of (p)ppGpp are regulated by RelA, a synthase, and SpoT, an enzyme with both synthase and hydrolase activities [1,4]. In other bacteria including *B*. *burgdorferi*, a single gene, *rel* or *rsh* (*rel/spo* homolog), encodes an enzyme with both synthase and hydrolase activities [6–8]. In those bacteria with a single *rel/rsh* ortholog, the N-terminal domain is responsible for the dual enzymatic activity while the C-terminal domain contains potential regulatory elements. Cytosolic levels of (p)ppGpp may also be controlled by other small GTPases [9]. Triggering of the stringent response by uncharged tRNA and activation and diffusion of ribosomal-bound RelA generates (p)ppGpp and leads to global changes in gene expression and intermediary metabolism [1–4]. These include decreased synthesis of stable rRNA and tRNA, proteolysis of ribosomal proteins, increased synthesis of amino acids, inhibition of motility, activation of *rpoN*-*rpoS* regulons and changes in carbon source utilization [1–3,10–13]. The net result is a shift to a slow- or non-growing state. Once stresses triggering the stringent response are removed, the short half-life of RelA/Rel and (p)ppGpp facilitates renewed synthesis of macromolecules and resumption of growth [1–4]. The ability of (p)ppGpp to shift transcription depends on its interaction with RNA polymerase, directing transcription from σ^70^ promoters to alternative promoters [1–3,13,14] often in synergy with the small regulator, DksA [11,12,15–17], as well as interactions with other proteins and regulatory RNAs [18]. In *E*. *coli*, *relA* expression is also regulated by the carbon storage regulator CsrA [19].

The stringent response is involved in bacterial virulence at multiple levels, having been shown to facilitate survival of extracellular pathogens in the host, transmissibility and persistence of a variety of intracellular pathogens [18,20,21], production of toxins [22], and host-vector cycling of vector-transmitted pathogens [23,24]. It also appears to be involved in the development of antimicrobial tolerance in bacteria by increasing the number of persister cells in culture [25–27]. Mutants of pathogens unable to produce (p)ppGpp are generally attenuated and have been proposed as live vaccines [24,28,29], while compounds able to block the production of (p)ppGpp may have therapeutic potential [30].

The life cycle of *Borrelia burgdorferi* sensu lato depends on its survival in several tissues and organs of ixodid tick vectors and mammalian reservoirs where it is exposed to challenging, variable and rapidly shifting availability of a range of nutrients [31,32]. The two component system-triggered regulatory pathway composed of Hk1/Rrp1/c-di-GMP increases borrelial survival during the larval and nymphal blood meals [33,34]. This pathway stimulates *B*. *burgdorferi* glycerol metabolism and together with genes of the *glp* operon is crucial for maximum fitness of the bacterium under these conditions [35,36]. Initial survival in mammalian reservoirs, in contrast, predominantly involves the Rrp2/RpoN(BosR)/RpoS cascade [33,34]. Linkage of other potential global regulators to these regulatory loops in *Borrelia* spp. (e.g., the stringent response, the carbon storage regulator protein CsrA) is still being characterized [37–43].

*B*. *burgdorferi* B31 contains a single chromosomal *rel* gene, *rel*_*Bbu*_ (BB0198, nt195693–197696), orthologous to *E*. *coli relA* and *spoT* [38,44,45]. Cloned *rel*_*Bbu*_ transcribed from its own promoter produced *rel*_*Bbu*_ mRNA and Rel_Bbu_ protein [37], was the only source of (p)ppGpp production in *B*. *burgdorferi* [37,38], and could complement an *E*. *coli relA/spoT* double null mutant [37]. The stringent response in *B*. *burgdorferi* was ameliorated during in vitro growth in the presence of tick cells and in engorged ticks [37,44]. Expression of *rel*_*Bbu*_ mRNA increased under in vitro conditions that presumably simulate the unfed tick state [46]. Amounts of Rel_Bbu_ were higher in *B*. *burgdorferi* growing in dialysis membrane chambers in vivo than in organisms growing in vitro despite similar levels of *rel*_*Bbu*_ mRNA under these two conditions [37]. A *B*. *burgdorferi rel*_*Bbu*_ null mutant (Δ*rel*_*Bbu*_) did not down-regulate synthesis of rRNA in stationary phase, a phenotype resembling that of a “relaxed” *E*. *coli* mutant, and was not infectious in mice [38,47].

In order to expand our understanding of the role of *rel*_*Bbu*_ and the stringent response in borrelial gene regulation and carbon source utilization, we have used *B*. *burgdorferi* microarrays to compare the global transcriptome in wild type *B*. *burgdorferi* 297 and its Δ*rel*_*Bbu*_ derivative during in vitro growth, and found the mutant to have a substantially altered transcriptome. Additional analyses confirmed that genes involved in glycerol utilization and metabolism are modulated by (p)ppGpp under these conditions [35,36].

## Results

### Effect of deletion of *rel*_*Bbu*_ on *B*. *burgdorferi* gene expression during exponential and stationary growth phases

The global alarmone nature of (p)ppGpp indicated that microarray analysis of wild type and Δ*rel*_*Bbu*_ strains would be useful for examining the role of Rel_Bbu_ and thus of (p)ppGpp in *B*. *burgdorferi* 297. From the multiplicity of growth conditions that could be chosen for these studies, we focused on the role of Rel_Bbu_ during in vitro growth at 34°C under two growth phases, exponential and stationary. Genes were considered to have altered expression between wild type and mutant strains if transcript levels differed by at least 2-fold at P ≤ 0.02. Increased expression of genes in the mutant relative to wild type implies (p)ppGpp-mediated repression of these genes in the wild type, whereas decreased expression is consistent with (p)ppGpp-mediated induction in the wild type. During the exponential phase of growth, 38 genes exhibited increased expression and 37 genes exhibited decreased expression in the mutant as compared to the parental *B*. *burgdorferi* wild type strain (S1 Table). The Δ*rel*_*Bbu*_ phenotype was more evident during stationary phase: 174 genes showed increased expression and 103 showed decreased expression in comparison to the wild type strain (S2 Table). Nineteen of 38 genes with higher expression in the exponential phase in *B*. *burgdorferi* Δ*rel*_*Bbu*_ were also elevated in stationary phase; 25 of 37 genes with lower expression in exponential phase in the mutant were also lower in stationary phase. These findings indicate that Rel_Bbu_ regulates expression of these genes in a similar manner in both exponential and stationary growth phases (S1 Table, S2 Table), and are also consistent with the production of (p)ppGpp during both growth phases in vitro [48,49]

**Fig 1:**
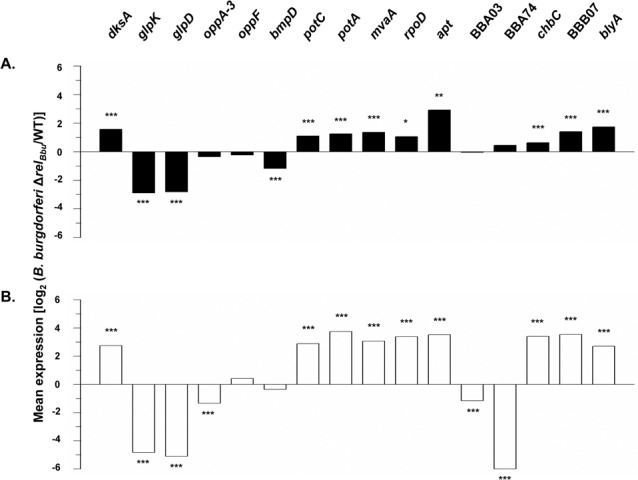
Transcriptional analysis by qRT-PCR of selected genes in *B*. *burgdorferi* 297 Δ*rel*_*Bbu*_ relative to expression of these genes in the wild type parental strain during (A) exponential and (B) stationary phases of growth in BSK-H at 34°C. *, P < 0.02; **, P < 0.005; ***, P < 0.001. Genes selected either had an assigned function and were regulated in operons, were involved in various aspects of cellular metabolism, were regulatory in nature or were localized to the cell envelope. Increased expression of genes in the mutant relative to wild type is consistent with *rel*-mediated repression of these genes in the wild type, while decreased expression is consistent with *rel*-mediated induction in the wild type. RT-PCR data were discordant with microarrays for *potA*, *mvaA*, *rpoD*, *apt*, BBB07, and *blyA* in the exponential phase of growth, and for *oppF* in the stationary phase of growth. See Materials and Methods for details of transcriptional analyses.

For validation of the microarray results, 16 genes (11 chromosomal, 5 plasmid) were selected for analysis by quantitative reverse transcription real-time PCR (RT-PCR) (Fig. 1). These genes were chosen on the basis of assigned functions: transcriptional regulators (*dksA*, *rpoD*); metabolism/transporters (*glpK*, *glpD*, *mvaA*, *apt*, *oppA-3*, *oppF*, *potC*, *potA*, *chbC*); cell envelope/membrane proteins (*bmpD*, BBA03, BBA74, BBB07, *blyA*). Expression levels of mRNA determined by quantitative RT-PCR were consistent with results from microarrays for 10 of 16 genes in exponentially growing cells and for 15 of 16 genes in stationary phase cells (Fig. 1). They were discordant for *potA*, *mvaA*, *rpoD*, *apt*, BBB07, and *blyA* whose expression in the exponential phase was elevated in the mutant by RT-PCR but not by microarray analysis, and for *oppF* whose expression in the stationary phase was decreased in the mutant by microarray but not by RT-PCR analysis (Table 1, S1 Table, S2 Table, Fig. 1).

**Table 1:** Modulated selected genes with annotated function in *B*. *burgdorferi* 297 Δ*rel*_*Bbu*_ during growth in vitro at 34°C^a^.

		**Exponential phase**		**Stationary phase**	
**Gene**	**Description**	**Mean expression (log_2_ [Δ*rel*_*Bbu*_/WT])**	**P**	**Mean expression (log_2_ [Δ*rel*_*Bbu*_/WT])**	**P**
Transcriptional regulators					
BB0168	*dnaK* suppressor (*dksA*)	1.31	0.002	3.26	<0.001
BB0419	response regulatory protein (*rrp-1*)			1.42	0.001
BB0420	sensory transduction histidine kinase/response regulator (*hk1*)	2.65	0.001	2.39	<0.001
BB0712	RNA polymerase σ^70^ factor (*rpoD*)	1.05	0.013	2.19	<0.001
DNA synthesis/repair					
BB0022	Holliday junction DNA helicase (*ruvB*)			2.58	<0.001
BB0344	DNA helicase (*uvrD*)			2.79	0.006
BB0438	DNA polymerase III, β subunit (*dnaN*)	2.41	0.006	1.56	<0.001
BB0552	DNA ligase (*lig*)			2.64	0.010
BB0579	DNA polymerase III, α subunit (*dnaE*)			4.95	0.001
BB0710	DNA primase (*dnaG*), authentic frameshift	1.26	<0.001	1.33	<0.001
BB0836	excinuclease ABC, B subunit (*uvrB*)			2.35	<0.001
BB0837	excinuclease ABC, A subunit (*uvrA*)			1.04	0.003
Cell division					
BB0177	glucose inhibited division protein B (*gidB*)			3.81	0.007
BB0178	glucose inhibited division protein A (*gidA*)			3.07	0.005
BB0302	cell division protein (*ftsW*)			2.96	0.001
BB0781	GTP-binding protein (*obg*)	1.03	0.004	1.95	<0.001
BB0789	cell division protein (*ftsH*)			1.01	<0.001
Protein synthesis					
BB0229	ribosomal protein L31 (*rpmE*)			1.54	<0.001
BB0251	leucyl-tRNA synthetase (*leuS*)			3.41	<0.001
BB0514	phenylalanyl-tRNA synthetase, β subunit (*pheT*)			1.63	0.009
BB0615	ribosomal protein S4 (*rpsD*)			2.87	<0.001
BB0691	translation elongation factor G (*fus-2*)			0.96	0.001
BB0778	ribosomal protein L21 (*rplU*)			1.51	0.002
BB0780	ribosomal protein L27 (*rpmA*)			1.03	<0.001
Motility/chemotaxis					
BB0147	flagellar filament 41 kDa core protein (*flaB*)			-1.31	0.001
BB0181	flagellar hook-associated protein (*flgK*)			-1.14	0.010
BB0271	flagellar biosynthesis protein (*flhA*)			1.95	0.002
BB0578	methyl-accepting chemotaxis protein (*mcp-1*)			3.06	0.012
BB0668	flagellar filament outer layer protein (*flaA*)			-1.13	<0.001
BB0670	purine-binding chemotaxis protein (*cheW*-3)			2.03	0.011
BB0775	flagellar hook-basal body complex protein (*flhO*)			1.03	<0.001
Cell envelope					
BB0382^d^	basic membrane protein B (*bmpB*)	-1.02	0.012	-1.59	<0.001
BB0383	basic membrane protein A (*bmpA*)			-1.15	0.016
BB0385	basic membrane protein D (*bmpD*)	-2.93	0.007		
BBA15	outer surface protein A (*ospA*)			-1.81	0.001
BBA16	outer surface protein B (*ospB*)			-1.54	0.002
BBA60	surface lipoprotein P27			-5.07	<0.001
BBA74	membrane-associated periplasmic protein			-2.03	<0.001
BBB07	α3β1 integrin-binding protein	1.42	<0.001		
BBJ41	antigen P35, putative			-8.62	<0.001
BBM23^b^	holin (*blyA*)	1.74	<0.001	1.43	<0.001
BBN24^c^	holin (*blyB*)	2.99	<0.001	1.17	<0.001
Central metabolism/carbon source transporters					
BB0240	glycerol uptake facilitator (*glpF*)	-4.54	<0.001	-4.76	<0.001
BB0241	glycerol kinase (*glpK*)	-8.27	<0.001	-5.47	<0.001
BB0243	glycerol-3-phosphate dehydrogenase (*glpD*)	-6.52	<0.001	-3.79	<0.001
BB0328	oligopeptide ABC transporter, periplasmic oligopeptide-binding protein (*oppA*-1)	-1.16	0.001	-2.65	0.002
BB0329	oligopeptide ABC transporter, periplasmic oligopeptide-binding protein (*oppA*-2)			-1.33	<0.001
BB0330	oligopeptide ABC transporter, periplasmic oligopeptide-binding protein (*oppA*-3)			-4.07	0.015
BB0334	oligopeptide ABC transporter, ATP-binding protein (*oppD*)	-3.25	0.006	-1.21	0.002
BB0335	oligopeptide ABC transporter, ATP-binding protein (*oppF*)			-1.51	<0.001
BBB04	chitobiose transporter protein (*chbC*)	1.83	0.004	2.11	<0.001
BBB05	chitobiose transporter protein (*chbA*)	4.05	0.013	5.07	<0.001
BBB06	chitobiose transporter protein (*chbB*)			5.02	<0.001
BB0683	3-hydroxy-3-methylglutaryl-CoA synthase (*hmgs*)	1.23	0.010	1.65	<0.001
BB0685	3-hydroxy-3-methylglutaryl-CoA reductase (*mvaA*)	1.36	<0.001	4.39	<0.001

a. Transcriptional analysis from microarrays (regular font) or RT-PCR (boldface). Where data from RT-PCR is shown, microarrays showed no significant difference in gene expression between *B*. *burgdorferi* 297 Δ*rel*_*Bbu*_ and wild type.

b. Expression values for *blyA* orthologs BBM23, BBP23, BBR23 that showed increased expression in stationary phase and BBN23, BBR23 and BBS23 that showed increased expression in exponential phase were considered as a single transcript because they are 100% identical in sequence.

c. Expression values for *blyB* orthologs BBN24, BBR24, BBS24 that showed increased expression in stationary phase and BBN24, BBR24, and BBS23 that showed increased expression in exponential phase were considered as a single transcript because they are 100% identical in sequence.

### *B*. *burgdorferi* Rel_Bbu_ regulon in exponential and stationary growth phases

More than 50% of the genes showing changes in transcriptional levels by microarray analysis (S1 Table, S2 Table) are annotated as hypothetical proteins and have no predicted biological role. This made functional analysis difficult. Genes with annotated functions in the *B*. *burgdorferi* Rel_Bbu_ regulon included those encoding transcriptional regulators or proteins involved in DNA synthesis and repair, cell division, protein synthesis, motility and chemotaxis, cell envelope synthesis, central metabolism and carbon source transport (Table 1, Fig. 1). These are all functions regulated by the stringent response in *E*. *coli* and other bacteria [1,12,50]

Several important transcriptional regulators were induced as a result of *rel*_*Bb*u_ deletion. The stringent response regulator *dksA* (BB0168) and σ^70^ (*rpoD*, BB0712) were upregulated in the Δ*rel*_*Bbu*_ mutant in both exponential and stationary growth phases (Table 1, Fig. 1). Genes coding for the two-component regulatory system 1 were also upregulated in the Δ*rel*_*Bbu*_ mutant. Expression of *rrp1* (BB0419) increased in stationary phase while expression of sensory histidine kinase *hk1* (BB0420) increased in both growth phases (Table 1), indicating that expression of these genes is repressed in the presence of (p)ppGpp. Neither *rpoN* nor *rpoS* was modulated by the stringent response in *B*. *burgdorferi*. This lack of effect of the *B*. *burgdorferi* stringent response on RpoS and OspC expression had been previously reported [38].

The *rel* mutant exhibited altered expression for genes encoding proteins involved in central metabolism and transport of carbon sources. Transcripts for two of the three subunits of the chitobiose transporter, *chbC* (BBB04) and *chbA* (BBB05), were elevated in the mutant during exponential phase and all three subunits (including *chbB*, BBB06) were elevated during stationary phase (Table 1, Fig. 1). In contrast, expression of the *glp* operon (BB0240-BB0243), encoding proteins involved in glycerol transport and metabolism were diminished in the *rel*_*Bbu*_ mutant (Table 1, Fig. 1). Genes encoding components of the oligopeptide ABC transporter, *oppA* (BB0328) and *oppD* (BB0334) were decreased in the *rel* mutant during the exponential phase, while many more of the genes of this transporter (BB0328-BB330, BB0334, BB0335) decreased in the mutant during stationary phase (Table 1, S1 Table, S2 Table). Expression of HMG-CoA synthase (*hmgs*, BB0683) was increased in the *rel* mutant during both exponential and stationary phase and HMG-CoA reductase (*mvaA*, BB0685) expression was increased during stationary phase (Table 1, Fig. 1), indicating that (p)ppGpp repression of the mevalonate pathway is part of the stringent response in *B*. *burgdorferi* [51].

### Effect of glucose and glycerol on growth of *B*. *burgdorferi rel*_*Bbu*_ mutant and derivatives

**Fig 2:**
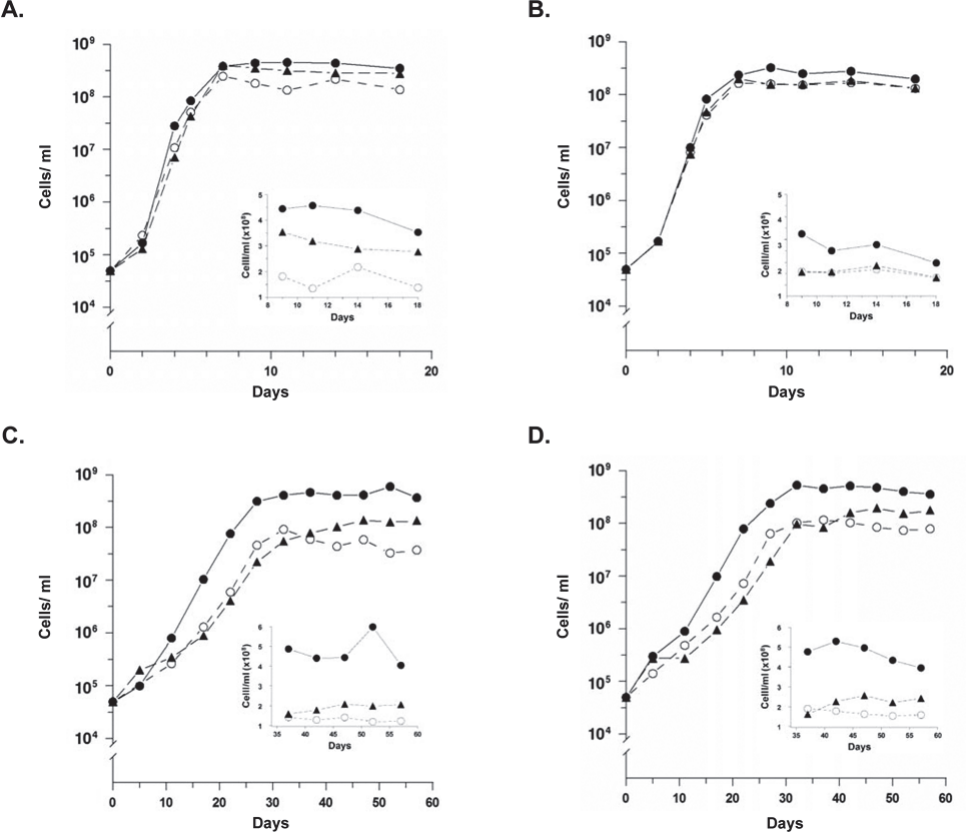
Growth of wild type *B*. *burgdorferi* 297 (solid circles), Δ*rel*_*Bbu*_ (open circles), or Δ*rel*_*Bbu*_ complemented with pKFSS1-Δ*rel*_*Bbu*_ (solid triangles) in BSK-Lite containing either glucose or glycerol as principal carbon sources. Insets show enlargement of cell concentrations for clarity. Glucose-containing medium, 34°C (A). Differences between stationary phase cell concentrations of wild type and Δ*rel*_Bbu_ mutant are significant as are differences in stationary phase concentrations between the Δ*rel*_Bbu_ mutant and its complemented derivative (P < 0.001, one-way ANOVA, Bonferroni post-test). Glycerol-containing medium, 34°C (B). Differences between stationary phase cell concentrations of wild type and Δ*rel*_Bbu_ mutant are significant (P < 0.001, one-way ANOVA, Bonferroni post-test). Differences in stationary phase concentrations between the Δ*rel*_Bbu_ mutant and its complemented derivative are not significant (P > 0.05, one-way ANOVA, Bonferroni post-test). Glucose-containing medium, 25°C (C). Differences between stationary phase cell concentrations of wild type and Δ*rel*_Bbu_ mutant are significant as are differences in stationary phase concentrations between the Δ*rel*_Bbu_ mutant and its complemented derivative (P < 0.001, one-way ANOVA, Bonferroni post-test). Glycerol-containing medium, 25°C (D). Differences between stationary phase cell concentrations of wild type and Δ*rel*_Bbu_ mutant are significant (P < 0.001, one-way ANOVA, Bonferroni post-test) as are differences in stationary phase concentrations between the Δ*rel*_Bbu_ mutant and its complemented derivative (P < 0.05, one-way ANOVA, Bonferroni post-test).

Microarray analysis strongly suggested that the lack of (p)ppGpp in the *rel*_*Bbu*_ deletion mutant reduced expression of the *glp* operon. Because glycerol and glucose are among the limited number of carbohydrates that can support *B*. *burgdorferi* growth [52–54], we investigated the ability of *B*. *burgdorferi* and its *rel* deletion mutant to utilize glycerol in BSK-Lite medium containing glycerol as the principal carbon source. The *B*. *burgdorferi* 297 parental wild type strain reached significantly higher cell concentrations during stationary phase than did the Δ*rel*_*Bbu*_ derivative during growth at either 25°C or 34°C in BSK-Lite containing glucose as the principal carbon source (P < 0.001, one-way ANOVA, Bonferroni post-test) (Fig. 2A and 2C). This impairment was partially reversed in the complemented strain (P < 0.001) (Fig. 2). These data are in agreement with earlier studies using BSK-H media containing glucose as the principal carbon source [38].

During growth at 34°C in BSK-Lite with glycerol as the principal carbon source, the wild type strain reached significantly higher cell concentrations in the stationary phase than the Δ*rel*_*Bbu*_ derivative and was not affected by complementation with the extrachromosomally-located gene (P < 0.01, one-way ANOVA, Bonferroni post-test) (Fig. 2B). During growth at 25°C in BSK-Lite containing glycerol, the wild type strain also reached a significantly higher cell density in the stationary phase than the Δ*rel*_*Bbu*_ mutant (P < 0.001, one-way ANOVA, Bonferroni post-test). The difference in cell concentrations between wild type *B*. *burgdorferi* and the *rel*_*Bbu*_ mutant was more obvious at 25°C than at 34°C with either glucose or glycerol suggesting a more important role for the stringent response in metabolic regulation at 25°C than at 34°C.

### Transcriptional analysis of *rel*_*Bbu*_ and the *glp* operon in *B*. *burgdorferi rel*_*Bbu*_ mutant and derivatives

**Fig 3:**
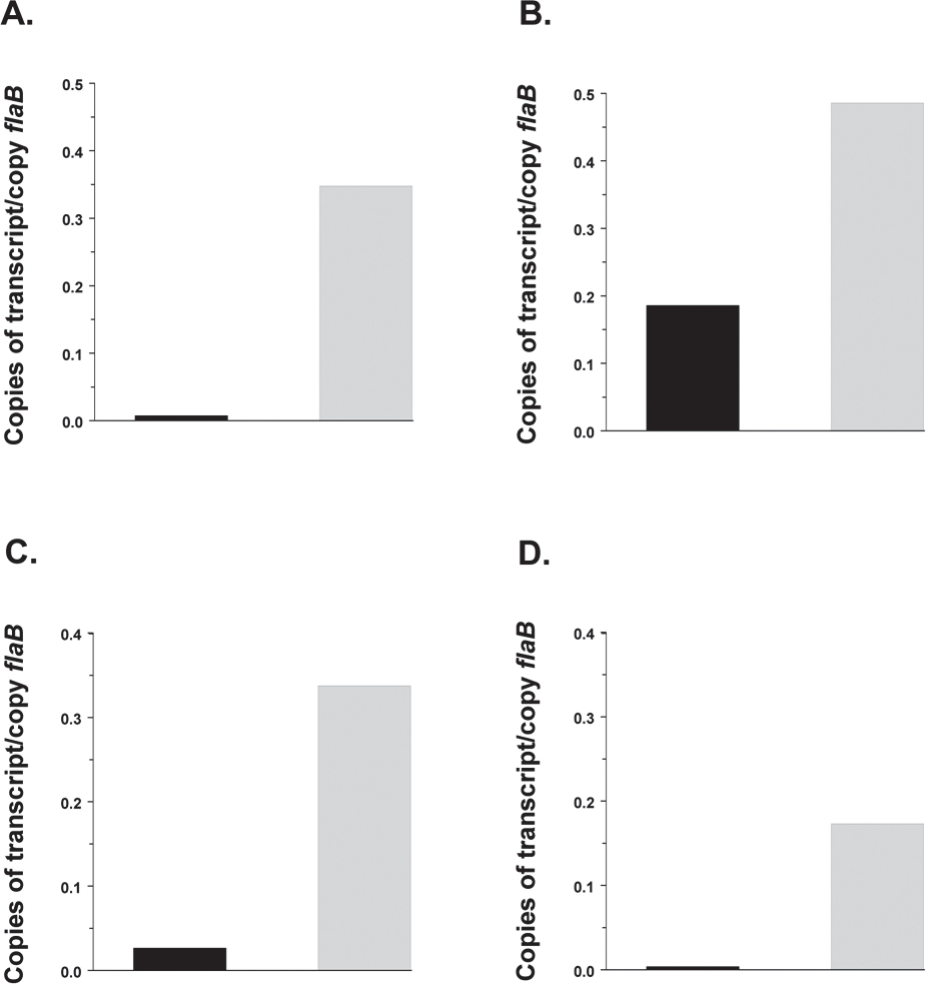
Transcriptional analysis of *rel*_*Bbu*_ in wild type *B*. *burgdorferi* 297 (solid bars), Δ*rel*_*Bbu*_ (open bars), and Δ*rel*_*Bbu*_ complemented with pKFSS1-Δ*rel*_*Bbu*_ (grey bars) during logarithmic growth in BSK-Lite medium containing glucose or glycerol as principal carbon sources. No bar is visible for the Δ*rel*_*Bbu*_ mutant because its expression level was zero. Glucose-containing medium, 34°C (A). Glycerol-containing medium, 34°C (B). Glucose-containing medium, 25°C (C). Glycerol-containing medium, 25°C (D).

**Fig 4:**
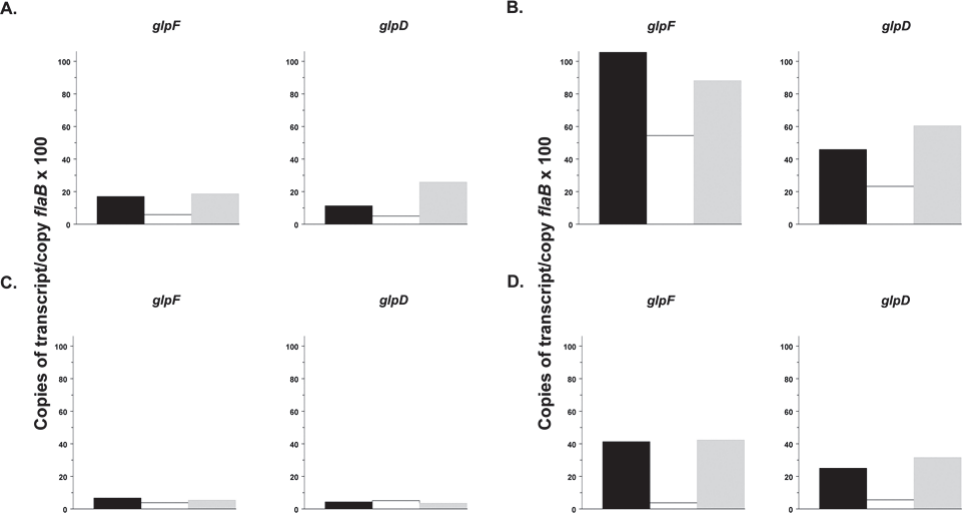
Transcriptional analysis of *glp* operon genes in wild type *B*. *burgdorferi* 297 (solid bars), Δ*rel*_*Bbu*_ (open bars), and Δ*rel*_*Bbu*_ complemented with pKFSS1-Δ*rel*_*Bbu*_ (grey bars) during late logarithmic growth phase in BSK-Lite medium containing glucose or glycerol as principal carbon sources. Glucose-containing medium, 34°C (A). Glycerol-containing medium, 34°C (B). Glucose-containing medium, 25°C (C). Glycerol-containing medium, 25°C (D).

The impairment of growth experienced by the Δ*rel*_*Bbu*_ mutant, coupled with the transcriptome data indicating reduced expression of the *glp* operon in the mutant, suggested that the stringent response might play a role in glycerol utilization in *B*. *burgdorferi*, particularly at 25°C. Transcriptional analysis in temperature-shifted organisms during logarithmic growth of wild type and Δ*rel*_*Bbu*_ mutant strains at 34°C and 25°C confirmed that *rel*_*Bbu*_ was not expressed in the deletion mutant and that expression of *rel*_*Bbu*_ at 34°C was greater in wild type cells growing on glycerol than in cells growing on glucose (Fig. 3). It also confirmed the role of (p)ppGpp in modifying transcription of glycerol metabolism genes. Transcript levels for the glycerol uptake facilitator (*glpF*, BB0240) and glycerol-3-phosphate dehydrogenase (*glpD*, BB0243), the first and the last genes in the *glp* operon, were substantially higher in wild type *B*. *burgdorferi* and in complemented Δ*rel*_*Bbu*_ mutant grown in media with glycerol as principal carbon source than in cells grown at 34°C in media with glucose as principal carbon source (Fig. 4A, 4B). Under both conditions, transcription of these genes was considerably lower in the Δ*rel*_*Bbu*_ mutant compared to the wild type or complemented strains (Fig. 4A, 4B). At 25°C, there was essentially no transcription of *glpD* and *glpF* in wild type or mutant *B*. *burgdorferi* grown in medium with glucose as principal carbon source (Fig. 4C). In contrast, these genes were actively transcribed in wild type and *rel*_*Bbu*_ complemented *B*. *burgdorferi* strains, but not in the Δ*rel*_*Bbu*_ mutant during growth in medium with glycerol as principal carbon source at this temperature (Fig. 4D). These changes in transcriptional levels of *glp* operon genes are expected to correlate with changes in levels of *glp* gene products, as we previously determined with *glpD* [36] and data not shown.

### Co-regulation of gene expression by stringent response and other global regulators in *B*. *burgdorferi*

To determine if the stringent response regulates glycerol uptake in *B*. *burgdorferi* indirectly through transcriptional cross talk with other global regulators, transcript levels of *rpoS*, *rpoN*, *bosR*, *rrp-1* and *rrp-2* were determined in wild type *B*. *burgdorferi* and its *rel*_*Bbu*_ derivatives during temperature-shifted exponential growth at 25°C and 34°C in media with glucose or glycerol as principal carbon sources. There were no significant differences in transcription of any of these genes between the wild type and the *rel*_*Bbu*_ mutant during exponential growth in either medium at either temperature (data not shown), similar to results from microarrays derived from the non-temperature-shifted exponential growth of *B*. *burgdorferi* at 34°C (Table 1, S1 Table).

## Discussion

The (p)ppGpp-mediated stringent response is a broadly conserved global regulatory response in bacteria that acts to preserve critical amounts of intracellular molecules in response to nutritional scarcity and/or other environmental stressors [1–3,5,48,50,55] such as those faced by *B*. *burgdorferi* in the distinctive vector and mammalian host environments it encounters in the course of its life cycle. We previously demonstrated that the *B*. *burgdorferi* stringent response could control growth, rRNA accumulation and virulence [38,47]. Microarray analysis validated by direct examination of gene expression of selected genes has shown that deletion of *rel*_*Bbu*_ and the subsequent lack of (p)ppGpp affects transcription of many genes in *B*. *burgdorferi* (75 during the exponential growth phase, 277 during stationary phase), and have confirmed production of this alarmone during both exponential and stationary growth phases [49]. The greater effects of (p)ppGpp during stationary phase were to be expected on the basis of our previous work showing that the lack of synthesis of (p)ppGpp preferentially affected borrelial growth and rRNA accumulation during transition from exponential growth to stationary phase and in the stationary phase itself [38,47]. The subsequent more detailed characterization of genes coding for glycerol transport and metabolism in the present study confirmed the regulation of glycerol metabolism in *B*. *burgdorferi* by the stringent response, a regulation likely crucial for its optimal growth in ticks.

Genes whose transcription was affected by deletion of *rel*_*Bbu*_ included those associated with DNA synthesis and repair, protein synthesis, cell motility and chemotaxis, cell wall synthesis and carbohydrate intermediary metabolism (Table 1, S1 Table, S2 Table). If genes with greater expression in the *rel*_*Bbu*_ deletion mutant than in the parental wild type are assumed to be repressed by (p)ppGpp (or other regulatory factors whose expression requires the presence of (p)ppGpp) and those with reduced expression in the *rel*_*Bbu*_ mutant are assumed to be induced in the presence of (p)ppGpp (or other regulatory factors whose synthesis is stimulated by (p)ppGpp), the stringent response in *B*. *burgdorferi* shares many similarities with this response in other bacteria [2,3,5,6,48,55–59]. The stringent response classically depresses motility and chemotaxis to save energy, and together with DksA, acts especially in stationary phase [11,12]. Modulation of borrelial motility by the stringent response may be particularly relevant in ticks where *B*. *burgdorferi* undergo temporal and spatial starvation and must migrate from the gut to the salivary glands to complete the enzootic cycle [60,61].

Despite these similarities, the stringent response in *B*. *burgdorferi* is not identical to that of other bacteria (Table 1, S1 Table, S2 Table). This is not surprising, given the phylogenetic distance between spirochetes and, for example, gammaproteobacteria such as *E*. *coli* [62]. While the stringent response in *E*. *coli* modulates expression of *rpoN* and *rpoS* [10,11,63], the stringent response in *B*. *burgdorferi* does not. Interestingly, comparison of microarray studies of null mutants of *rel*/(p)ppGpp (this study) and *rpoS* [64] uncovered 17 genes that were modulated in both studies (Table 2). Fifteen of these 17 genes were repressed by (p)ppGpp only in the stationary phase, while all 17 were activated by RpoS. This might suggest that the effects of (p)ppGpp and RpoS on gene expression in *B*. *burgdorferi* during in vitro growth are independent of, or counterbalance, each other. Unfortunately, comparison between the in vitro stringent response regulon in cells at stationary phase and the RpoS regulon in exponentially growing cells may not be relevant, particularly since both microarrays and quantitative RT-PCR found no significant differences in transcript levels between these and other global regulators in *B*. *burgdorferi* wild type and *rel*_*Bbu*_ mutant during exponential growth at 34°C in vitro. While several genes induced by (p)ppGpp (the *glp* operon, BB365, *ospA/B*, BBA62, BBA69, BBA74, BBD18, BBJ41) during stationary phase in vitro were repressed by RpoS under mammalian host-like conditions [64], the difference between these two culture conditions again makes evaluation of any potential interplay between these two transcriptional regulators uncertain.

**Table 2:** Genes modulated during growth *in vitro* in *B*. *burgdorferi* Δ*rel*_*Bbu*_ (S2 Table) and *B*. *burgdorferi* Δ*rpoS* [64].

		Mean expression	
Gene	Description	log_2_ (Δ*rel*_*Bbu*_/WT)	log_2_ (Δ*rpoS*_*Bbu*_/WT)
BB0670	purine-binding chemotaxis protein (*cheW-3*)	2.03	-2.56
BB0782	nicotinate (nicotinamide) nucleotide adenylyltransferase	2.99	-2.88
BBA12	conserved hypothetical protein	2.81	-2.73
BBA60	surface lipoprotein P27	-5.07	-2.58
BBM01	hypothetical protein	3.57	-3.05
BBM08	conserved hypothetical protein	3.04	-2.76
BBN01	hypothetical protein	3.82	-2.85
BBN29	hypothetical protein, paralogous family 161, authentic point mutation	3.07	-4.62
BBO03	hypothetical protein	3.91	-2.59
BBO04	hypothetical protein	3.08	-2.85
BBO29	hypothetical protein	2.54	-3.30
BBP21	conserved hypothetical protein	2.14	-2.81
BBP25	conserved hypothetical protein	4.83	-3.28
BBP28	Lipoprotein	-3.07	-3.72
BBR02	hypothetical protein, paralogous family 147, authentic frameshift	4.36	-3.31
BBR29	conserved hypothetical protein	2.83	-7.17
BBS01	hypothetical protein	4.22	-3.14

Additional differences between the stringent response in *B*. *burgdorferi* and that of other bacteria result from genes modulated by the stringent response that are unique to this bacterium (e.g., genes for antigenic surface proteins, *bly* genes) [52,65,66]. Regulation of these genes by the stringent response could influence the composition of the *B*. *burgdorferi* cell envelope to provide a surface better able to endure variable environments such as those encountered in the tick [34]. The ability of the stringent response to modulate peptidoglycan synthesis through its modulation of the mevalonate pathway could also result in antigenic alterations and round forms [67]. Existence of these changes could suggest that niche nutritional signals can be coupled to cell surface antigenic composition and functionality to develop interactions that favor *B*. *burgdorferi* residence [34,60,61].

Stringent response-mediated regulation of genes involved in nutrient transport and metabolism is common in *E*. *coli* and many other bacteria [1,5]. *B*. *burgdorferi* lacks amino acid biosynthetic pathways [52] and is dependent on oligopeptide uptake to satisfy its requirement for amino acids. Transcriptional modulation of the oligopeptide transporter system by the stringent response observed in the present study expands the role of (p)ppGpp to this essential borrelial function [68,69]. Expression of the *glp* operon (Table 1) was inhibited in the *rel* mutant in both exponential and stationary growth phases, suggesting that transcription of this operon is induced by (p)ppGpp. In contrast, transcription of genes encoding the three components of the chitobiose phosphotransferase system transporter located on plasmid cp26 (Table 1) was elevated in the *rel* mutant in the exponential and stationary phase of growth, suggesting that their transcription is directly or indirectly repressed by (p)ppGpp. Alterations in expression of genes involved in utilization of carbon sources such as glycerol and chitobiose strongly suggest that the borrelial stringent response plays an important role in modifying synthesis of macromolecules, central metabolism and carbon utilization in ticks [2,50,55].

Previous reports of regulation of *glp* operon expression by RpoS and Rrp1 [35,64], and the essential role of glycerol uptake and metabolism for maximum *B*. *burgdorferi* fitness in ticks [35,36] prompted a more detailed examination of the regulation of this operon by the stringent response and (p)ppGpp. Decreased ability of the *rel*_*Bbu*_ mutant to grow in medium with glycerol as the principal carbon source (Fig. 2B, 2D) and repression of transcription of glycerol metabolism genes at 25°C compared to 34°C during exponential growth (Fig. 4B, 4D) is similar to a phenotype previously described for a *B*. *burgdorferi rrp1* mutant that cannot synthesize c-di-GMP [35]. This could suggest that the stringent response controls glycerol uptake by down-modulating Rrp1 in *B*. *burgdorferi* at 25°C in ticks. Because (p)ppGpp repressed *rrp1* in stationary phase and induced expression of the *glp* operon in both exponential and stationary phases at 34°C (Table 1), one would expect to see increased c-di-GMP and increased transcription of glycerol metabolism genes in the *rel*_*Bbu*_ mutant rather than the observed decrease. This could suggest that despite down-modulation of Rrp1 expression by (p)ppGpp, sufficient Rrp1 remains to allow production of c-di-GMP. This might indicate that c-di-GMP and (p)ppGpp act synergistically to increase expression of the *glp* operon in both exponential and stationary phases (Table 1), and are both necessary for development of optimal regulatory activity. Alternatively, synthesis of c-di-GMP by Rrp1 might be dependent on the presence of (p)ppGpp [70]. In any case, these data suggest that (p)ppGpp adds an additional layer of control to the regulation of *glp* operon expression by Rrp1 [35]. The precise mechanism by which (p)ppGpp and c-di-GMP act to induce *glp* operon expression, and the inter-relationships of *rel*_*Bbu*_ and *rrp1* in modulating this expression, clearly warrant further study.

The greater expression of the *glp* genes at 34°C contradicts previous results where these genes were better expressed at 25°C than at 34°C [36]. Although there was little *rel*_*Bbu*_ transcript in the wild type at 25°C, it remains unclear how much Rel_Bbu_ activity is required for expression of glycerol utilization genes. The apparent discrepancy between the low expression of *rel*_*Bbu*_ and the *glp* genes at 25°C with strong growth of wild type *B*. *burgdorferi* 297 and its complemented derivative at this temperature could indicate that this amount of *rel*_*Bbu*_ expression produces adequate (p)ppGpp to maintain sufficient expression of *glpD* and *glpF* for growth under these conditions [49]. It could also result from strain differences, since different *B*. *burgdorferi* strains have been shown to exhibit differences in the stringent response [37,38].

Expression of the genes encoding components of the chitobiose PTS was elevated in the *rel*_*Bbu*_ mutant, implying that (p)ppGpp inhibits chitobiose uptake. In contrast to the *glp* operon which is regulated by RpoS, Rrp1 and BosR, *chbCAB* transporter genes are not members of any of these regulons [35,64,71]. However, a recent study did suggest that *chbC* expression was significantly lower in an Rrp1 mutant not expressing RpoS or BosR [72]. While this implies that either RpoS or BosR are required for minimal expression of *chbC* and is consistent with the findings of Rhodes et al. [73], it is not consistent with those of Pappas et al [36]. These latter workers found that *chbC* transcripts were significantly higher in ticks than in mouse joints [36]. Although a *chbC* mutant could not utilize chitobiose, it could successfully complete the mouse-tick infectious cycle, suggesting that chitobiose utilization is dispensable for this latter process [74]. Resolution of these apparently contradictory findings and the possible role of (p)ppGpp in modulating chitobiose uptake will require further research.

The relationship between glycerol and chitobiose utilization is complex. Modulation of genes involved in utilization of glycerol and chitobiose by global regulators is likely to be relevant for survival of *B*. *burgdorferi* in ticks [35,36,72–75]. The ability of *B*. *burgdorferi* to utilize glycerol in unfed nymphs may in fact be essential for effectively surviving the non-replicative quiescent state [76]. In other bacteria, the stringent response and glycerol metabolism are associated with establishing and maintaining the persistent state [76,77]. The data presented here could suggest that a similar situation is operative in *B*. *burgdorferi*.

Although both the *glp* operon and chitobiose transporter genes are expressed at significantly higher levels in ticks than in mammals [36], the regulatory mechanisms controlling utilization of the two carbohydrates are distinct. Repression of *glp* operon expression during the mammalian phase is RpoS-dependent, but this has not been definitively demonstrated for *chbC*. Elevation of transcripts for all components of the chiotobiose transporter in the *rel*_*Bbu*_ mutant suggests that (p)ppGpp might be the regulatory molecule responsible for expression of the chitobiose transport genes. Further studies of *B*. *burgdorferi* growing under in vivo conditions throughout the enzootic cycle will be necessary to clarify the potential contribution of *rel* and (p)ppGpp to the regulation of glycerol and chitobiose utilization.

It was previously suggested that *B*. *burgdorferi glp* mutants survived in flat and feeding nymphs because they had access to chitobiose that became available during dissolution of the peritrophic matrix and molting [36]. It could be hypothesized that the stringent response in *B*. *burgdorferi* links nutritional stress with sequential utilization of hexoses, glycerol and chitobiose [1,50,53,55]. The role played by (p)ppGpp in this sequential utilization during shifts from exponential phase in feeding larvae to stationary phase in flat nymphs and again to exponential growth in feeding nymphs is consonant with the role this alarmone plays in other bacteria, and is comparable to diauxic growth in *E*. *coli* facing similar nutritional challenges [1,50,53,55].

There are several possible mechanisms by which (p)ppGpp could regulate gene expression in *B*. *burgdorferi*. It could directly affect transcription, or act indirectly by changing levels of other transcriptional regulators [2,3,19,48] or by modifying protein function by direct binding [78]. For example, we found that mRNA expression of two transcriptional regulators, DksA and Rrp1 was increased in the *rel*_*Bbu*_ deletion mutant relative to wild type (Table 1). While this is consistent with repression of expression of these genes by (p)ppGpp, it could also result from a mechanism where the absence of one of these regulators was compensated by overproduction of another [10,11,79]. In *E*. *coli*, an increase in DksA can compensate for the lack of (p)ppGpp and can act synergistically, antagonistically and independently of (p)ppGpp to regulate mRNA transcription [80]. In other bacteria, DksA is required for negative regulation of rRNA and ribosomal protein expression and positive regulation of maintenance and stress-resistance promoters during the stringent response [17,81,82]. In the present case, our results suggest that DksA may act synergistically with (p)ppGpp in *B*. *burgdorferi*; further work is needed to determine whether there is a total or partial coincidence of the Rel and DskA regulons in this organism. (p)ppGpp does not seem to be involved in regulation of Rrp2, RpoN and BosR in *B*. *burgdorferi* since transcript amounts for the genes encoding these regulators were not affected by the deletion of *rel*_*Bbu*_.

Stringent response regulation of glycerol and chitobiose gene expression might be mediated directly at the level of transcription by interactions of (p)ppGpp and DksA with the RNA polymerase complex at the glycerol and chitobiose gene promoters [1,14,83]. That there were no differences in transcriptional levels of *rpoS*, *rpoN*, *bosR*, and *rrp2* between the *rel*_*Bbu*_ mutant and wild type *B*. *burgdorferi* under any experimental conditions examined suggests a direct interaction of (p)ppGpp with the RNA polymerase complexes at the *glp* and chitobiose operon promoters or interactions with still uncharacterized regulators [1,14,48,50,83]. The *B*. *burgdorferi* genome does not encode regulators such as SlyA and PigR that are directly involved in transcriptional regulation mediated by (p)ppGpp in other bacteria, but the role of DksA as a global regulator in *B*. *burgdorferi* has not yet been defined [1]. In *E*. *coli*, the CsrA and stringent response regulatory pathways have been shown to be linked [19]. The recently characterized *B*. *burgdorferi* CsrA and PlzA homologues could therefore be potential regulators responsible for linking the stringent response to Hk2-Rrp2/BosR-RpoN/RpoS and the Hk1-Rrp1-c-di-GMP cascades in their ability to regulate glycerol and chitobiose metabolism [19,39,43,84]. However, more recent work suggests that CsrA is not involved in regulation of the RpoN-RpoS axis in *B*. *burgdorferi* [85].

In summary, we have established the stringent response/(p)ppGpp regulon in *B*. *burgdorferi* during exponential and stationary phase growth in vitro. We show that (p)ppGpp stimulates induction of the *glp* operon and repression of chitobiose transporter gene expression. A limitation of the present findings is that they are based on expression analysis of *B*. *burgdorferi* during in vitro culture. Additional studies will be necessary to examine the nature of the stringent response in vivo in both ticks and mammals. Possible differences in the stringent response in *B*. *burgdorferi* strains with differing virulence potential may also warrant investigation.

## Materials and Methods

### *B*. *burgdorferi* strains and culture conditions

*B*. *burgdorferi* 297 (clone BbAH130) was kindly provided by Dr. M. V. Norgard, University of Texas Southwestern Medical Center. This strain was the parental strain for the *B*. *burgdorferi* Δ*rel*_*Bbu*_ deletion mutant and its complemented derivative, *B*. *burgdorferi* Δ*rel*_*Bbu*_ pKFSS1-*rel*_*Bbu*_ [38]. *B*. *burgdorferi* strains were maintained at 34°C in Barbour-Stoenner-Kelley (BSK)-H (Sigma-Aldrich, St. Louis, MO) supplemented with 6% heat-inactivated rabbit serum (Sigma). *B*. *burgdorferi* Δ*rel*_*Bbu*_ was grown in the presence of 400 μg/ml of kanamycin (Sigma), *B*. *burgdorferi* Δ*rel*_*Bbu*_ pKFSS1-*rel*_*Bbu*_ was grown in the presence of 400 μg/ml of kanamycin and 50 μg/ml of streptomycin (Sigma).

For microarray experiments, strains were grown continuously at 34°C, and were not temperature shifted [46]. For growth experiments and expression analysis of *rel*_*Bbu*_ and *glp* genes in modified growth medium with different carbon sources at different temperatures, *B*. *burgdorferi* 297 and Δ*rel*_*Bbu*_ derivatives were temperature shifted. The cells for each strain were grown to late log phase (5–10 x 10^7^ cells/ml) in BSK-II medium [86] at 25°C, and then diluted to a final concentration of 5 x 10^4^ in 40 ml of BSK-Lite with N-acetylglucosamine [36,53] and either 0.4% glucose or 0.4% glycerol as principal carbon source. Quadruplicate 5 ml aliquots of each strain with each principal carbon source were incubated at 25°C or 34°C for up to 60 days, or until one week after stationary phase was reached. Technical replicate tubes were counted daily (for cultures incubated at 34°C) or every two days (for cultures incubated at 25°C) by dark field microscopy.

### Microarray analysis

*B*. *burgdorferi* parental and Δ*rel*_*Bbu*_ cells were collected from duplicate cultures during exponential growth (5 x 10^7^ cells/ml for wild type, 2 x 10^7^ cells/ml for Δ*rel*_*Bbu*_) and stationary phase (4x10^8^ cells/ml for wild type, 8x10^7^ cells/ml for Δ*rel*_*Bbu*_) at 34°C. RNA was isolated using TRIzol (Invitrogen Life Technologies, Carlsbad, CA), treated with RQ1 RNase-free DNase (Promega Corporation, Madison, WI) and fluorescently labeled with Cy3 or Cy5 dye by reverse transcription. Microarray hybridizations were performed as described [64] with cDNA prepared from cells for each growth phase with two biological replicates and with two technical replicates (dye swap). Data acquired using GenePix software were transferred to Microsoft Excel for background subtraction and normalization [64]. Significance of differential expression was determined by two-tailed, unpaired Student *t* test at P<0.02 and fold-comparison >2. Genes located on plasmids of *B*. *burgdorferi* B31 that are printed on microarray slides, but are absent in *B*. *burgdorferi 297* [87] were removed from the final output. Microarray data have been submitted under ArrayExpress (https://www.ebi.ac.uk/arrayexpress), accession number E-MTAB-3029.

### Reverse transcription and real-time PCR

Two reverse transcription real-time PCR protocols were used in this work: one to validate the results of the microarrays of *B*. *burgdorferi* strains grown in exponential and stationary phases and the other to determine gene expression in *B*. *burgdorferi* strains growing in BSK-Lite.

To validate microarray results, cDNA was synthesized using 1 μg of total *B*. *burgdorferi* RNA isolated for microarray analysis, the primers listed in Table 2, and AMV reverse transcriptase (Promega) following the manufacturer’s instructions. The resulting cDNA was quantified by real-time PCR with primers specific for each gene (S3 Table) using the LightCycler Master SYBR Green I Mixture (Roche) and a LightCycler Real-time PCR instrument (Roche). To compare mRNA levels, PCR reactions were performed with both biological replicates used for microarray analysis. Each experimental sample was analyzed in duplicate. Genomic DNA from 10^3^–10^6^ cells of the corresponding *B*. *burgdorferi* strain was used as a standard to estimate the amount of cDNA of genes studied in each real-time PCR. Transcript amounts for each gene were normalized to cDNA of constitutively expressed *flaB*. Results are reported as log_2_ mean expression of relevant genes in *B*. *burgdorferi* Δ*rel*_*Bbu*_ relative to their expression in the parental wild type strain.

To determine gene expression in *B*. *burgdorferi* strains growing in BSK-Lite, RNA was isolated from late log phase cells growing at 34°C and 25°C using TRIzol and treated twice with the Ambion DNA *free* kit (Ambion, Austin, TX) according to the manufactuer’s instructions to remove DNA. cDNA was synthesized using 2 μg of purified RNA, random hexamer primers (Promega), and AMV reverse transcriptase enzyme (Promega). Real-time PCR reactions were performed as previously described [88] using primers listed in S3 Table. Copy number for *flaB* was determined for each biological sample and copy number for each gene was then normalized to copies of *flaB*.

### Data analysis

Statistical analysis of growth curves was performed on log_2_ transformed data using one-way ANOVA. Significance was defined as P ≤ 0.05. Data from microarrays were analyzed as previously described [64].

## Supporting Information

S1 Table Genes modulated in *B*. *burgdorferi* 297 Δ*rel*_*Bbu*_ during exponential phase of growth in vitro at 34°C by microarray analysis. (DOC)

S2 Table Genes modulated in *B*. *burgdorferi* 297 Δ*rel*_*Bbu*_ during stationary phase of growth in vitro at 34°C by microarray analysis. (DOC)

S3 Table Primers used in this study. (DOC)
