# Correction: Characterization of the Rel_Bbu_ Regulon in *Borrelia burgdorferi* Reveals Modulation of Glycerol Metabolism by (p)ppGpp

**DOI:** 10.1371/journal.pone.0123614

**Published:** 2015-03-30

**Authors:** The PLOS ONE Staff

There are formatting errors in Table 1. Please see the corrected Table 1 here:

**Table 1:** Modulated selected genes with annotated function in *B*. *burgdorferi* 297 Δ*rel*_*Bbu*_ during growth in vitro at 34°C^a^.

		Exponential phase		Stationary phase	
Gene	Description	Mean expression(log_2_ [Δ*rel*_*Bbu*_/WT])	P	Mean expression(log_2_ [Δ*rel*_*Bbu*_/WT])	P
**Transcriptional regulators**					
BB0168	*dnaK* suppressor (*dksA*)	1.31	0.002	3.26	<0.001
BB0419	response regulatory protein (*rrp-1*)			1.42	0.001
BB0420	sensory transduction histidine kinase/response regulator (*hk1*)	2.65	0.001	2.39	<0.001
BB0712	RNA polymerase σ^70^ factor (*rpoD*)	**1.05**	**0.013**	2.19	<0.001
**DNA synthesis/repair**					
BB0022	Holliday junction DNA helicase (*ruvB*)			2.58	<0.001
BB0344	DNA helicase (*uvrD*)			2.79	0.006
BB0438	DNA polymerase III, β subunit (*dnaN*)	2.41	0.006	1.56	<0.001
BB0552	DNA ligase (*lig*)			2.64	0.010
BB0579	DNA polymerase III, α subunit (*dnaE*)			4.95	0.001
BB0710	DNA primase (*dnaG*), authentic frameshift	1.26	<0.001	1.33	<0.001
BB0836	excinuclease ABC, B subunit (*uvrB*)			2.35	<0.001
BB0837	excinuclease ABC, A subunit (*uvrA*)			1.04	0.003
**Cell division**					
BB0177	glucose inhibited division protein B (*gidB*)			3.81	0.007
BB0178	glucose inhibited division protein A (*gidA*)			3.07	0.005
BB0302	cell division protein (*ftsW*)			2.96	0.001
BB0781	GTP-binding protein (*obg*)	1.03	0.004	1.95	<0.001
BB0789	cell division protein (*ftsH*)			1.01	<0.001
**Protein synthesis**					
BB0229	ribosomal protein L31 (*rpmE*)			1.54	<0.001
BB0251	leucyl-tRNA synthetase (*leuS*)			3.41	<0.001
BB0514	phenylalanyl-tRNA synthetase, β subunit (*pheT*)			1.63	0.009
BB0615	ribosomal protein S4 (*rpsD*)			2.87	<0.001
BB0691	translation elongation factor G (*fus-2*)			0.96	0.001
BB0778	ribosomal protein L21 (*rplU*)			1.51	0.002
BB0780	ribosomal protein L27 (*rpmA*)			1.03	<0.001
**Motility/chemotaxis**					
BB0147	flagellar filament 41 kDa core protein (*flaB*)			-1.31	0.001
BB0181	flagellar hook-associated protein (*flgK*)			-1.14	0.010
BB0271	flagellar biosynthesis protein (*flhA*)			1.95	0.002
BB0578	methyl-accepting chemotaxis protein (*mcp-1*)			3.06	0.012
BB0668	flagellar filament outer layer protein (*flaA*)			-1.13	<0.001
BB0670	purine-binding chemotaxis protein (*cheW*-3)			2.03	0.011
BB0775	flagellar hook-basal body complex protein (*flhO*)			1.03	<0.001
**Cell envelope**					
BB0382^d^	basic membrane protein B (*bmpB*)	-1.02	0.012	-1.59	<0.001
BB0383	basic membrane protein A (*bmpA*)			-1.15	0.016
BB0385	basic membrane protein D (*bmpD*)	-2.93	0.007		
BBA15	outer surface protein A (*ospA*)			-1.81	0.001
BBA16	outer surface protein B (*ospB*)			-1.54	0.002
BBA60	surface lipoprotein P27			-5.07	<0.001
BBA74	membrane-associated periplasmic protein			-2.03	<0.001
BBB07	α3β1 integrin-binding protein	**1.42**	<0.001		
BBJ41	antigen P35, putative			-8.62	<0.001
BBM23^b^	holin (*blyA*)	**1.74**	<0.001	1.43	<0.001
BBN24^c^	holin (*blyB*)	2.99	<0.001	1.17	<0.001
**Central metabolism/carbon source transporters**					
BB0240	glycerol uptake facilitator (*glpF*)	-4.54	<0.001	-4.76	<0.001
BB0241	glycerol kinase (*glpK*)	-8.27	<0.001	-5.47	<0.001
BB0243	glycerol-3-phosphate dehydrogenase (*glpD*)	-6.52	<0.001	-3.79	<0.001
BB0328	oligopeptide ABC transporter, periplasmic oligopeptide-binding protein (*oppA*-1)	-1.16	0.001	-2.65	0.002
BB0329	oligopeptide ABC transporter, periplasmic oligopeptide-binding protein (*oppA*-2)			-1.33	<0.001
BB0330	oligopeptide ABC transporter, periplasmic oligopeptide-binding protein (*oppA*-3)			-4.07	0.015
BB0334	oligopeptide ABC transporter, ATP-binding protein (*oppD*)	-3.25	0.006	-1.21	0.002
BB0335	oligopeptide ABC transporter, ATP-binding protein (*oppF*)			-1.51	<0.001
BBB04	chitobiose transporter protein (*chbC*)	1.83	0.004	2.11	<0.001
BBB05	chitobiose transporter protein (*chbA*)	4.05	0.013	5.07	<0.001
BBB06	chitobiose transporter protein (*chbB*)			5.02	<0.001
BB0683	3-hydroxy-3-methylglutaryl-CoA synthase (*hmgs*)	1.23	0.010	1.65	<0.001
BB0685	3-hydroxy-3-methylglutaryl-CoA reductase (*mvaA*)	**1.36**	<0.001	4.39	<0.001

^a^Transcriptional analysis from microarrays (regular font) or RT-PCR (boldface). Where data from RT-PCR is shown, microarrays showed no significant difference in gene expression between *B*. *burgdorferi* 297 Δ*rel*_*Bbu*_ and wild type.

^b^Expression values for *blyA* orthologs BBM23, BBP23, BBR23 that showed increased expression in stationary phase and BBN23, BBR23 and BBS23 that showed increased expression in exponential phase were considered as a single transcript because they are 100% identical in sequence.

^c^Expression values for *blyB* orthologs BBN24, BBR24, BBS24 that showed increased expression in stationary phase and BBN24, BBR24, and BBS23 that showed increased expression in exponential phase were considered as a single transcript because they are 100% identical in sequence.
