# Supplementary material for: Characterization of the RelBbu Regulon in Borrelia burgdorferi Reveals Modulation of Glycerol Metabolism by (p)ppGpp
Source: PLoS One. 2015 Feb 17;10(2):e0118063. doi: 10.1371/journal.pone.0118063 (PMC4331090; doi:10.1371/journal.pone.0118063)
Supplement: S1 Table — (DOC) [file pone.0118063.s001.doc]

| **Table S1.** Genes modulated in *B. burgdorferi* 297 Δ*rel_Bbu_* during exponential phase of growth in vitro at 34°C by microarray analysis | | | |
| --- | --- | --- | --- |
| **ID** | **Description** | **Mean expression** | **P** |
|  |  | **[log_2_ (Δ*rel_Bbu_*/WT)]** |  |
| **Genes showing increased expression (n=38 )** | | | |
| BBA23^a^ | conserved hypothetical protein | 4.59 | <0.001 |
| BBB05^a^ | PTS system, chitobiose-specific IIA component (*chbA*) | 4.05 | 0.013 |
| BB0640^a^ | spermidine/putrescine ABC transporter, permease protein (*potC*) | 3.91 | 0.001 |
| BBO02^a^ | hypothetical protein | 3.46 | 0.011 |
| BB0106 | TPR domain protein | 3.18 | 0.01 |
| BBN19 | conserved hypothetical protein | 3.07 | 0.006 |
| BBN24^a,c^ | hemolysin accessory protein (*blyB*) | 2.99 | <0.001 |
| BBN23^b^ | pore-forming hemolysin (*blyA*) | 2.91 | <0.001 |
| BBA38 | hypothetical protein | 2.87 | 0.003 |
| BBS21^a^ | conserved hypothetical protein | 2.77 | 0.005 |
| BB0420^a^ | sensory transduction histidine kinase/response regulator (*hk1*) | 2.65 | 0.001 |
| BB0429^a^ | hypothetical protein | 2.64 | 0.01 |
| BB0677 | ribose/galactose ABC transporter, ATP-binding protein (*mglA*) | 2.52 | <0.001 |
| BBA17 | hypothetical protein | 2.51 | 0.008 |
| BBK11 | hypothetical protein | 2.45 | 0.016 |
| BB0525^a^ | conserved hypothetical protein | 2.44 | 0.007 |
| BB0438^a^ | DNA polymerase III, β subunit (*dnaN*) | 2.41 | 0.006 |
| BB0434 | stage 0 sporulation protein J (*spo0J*) | 2.36 | 0.004 |
| BB0298 | TPR domain protein | 2.23 | 0.015 |
| BB0015 | uridine kinase (*udk*) | 2.18 | 0.018 |
| BBB04^a^ | PTS system, chitobiose-specific IIC component (*chbC*) | 1.83 | 0.004 |
| BBA22 | hypothetical protein | 1.74 | <0.001 |
| BBN20^a^ | conserved hypothetical protein | 1.71 | 0.01 |
| BB0201 | UDP-N-acetylmuramoylalanyl-D-glutamate-2,6-diaminopimelate ligase (*murE*) | 1.62 | 0.018 |
| BB0726^a^ | *minD*-related ATP-binding protein (*ylxH-3*) | 1.61 | 0.015 |
| BB0295 | heat shock protein (*hslU*) | 1.49 | 0.02 |
| BB0441^a^ | ribonuclease P protein component (*rnpA*) | 1.38 | 0.02 |
| BB0168^a^ | *dnaK* suppressor (*dksA*) | 1.31 | 0.002 |
| BB0776^a^ | hypothetical protein | 1.29 | 0.007 |
| BB0710^a^ | DNA primase (*dnaG*), authentic frameshift | 1.26 | <0.001 |
| BBS19 | conserved hypothetical protein | 1.26 | 0.009 |
| BB0683^a^ | 3-hydroxy-3-methylglutaryl-CoA synthase (*hmgs*) | 1.23 | 0.01 |
| BB0190 | translation initiation factor 3 (*infC*) | 1.15 | 0.001 |
| BB0711^a^ | hypothetical protein | 1.10 | 0.007 |
| BB0709 | aminodeoxychorismate lyase, putative (COG1559) | 1.04 | <0.001 |
| BB0781^a^ | GTP-binding protein (*obg*) | 1.03 | 0.004 |
| BB0343^a^ | Glu-tRNA(Gln) amidotransferase, subunit C (*gatC*) | 1.02 | 0.01 |
| BBN13 | hypothetical protein, paralogous family 154, authentic frameshift | 1.00 | 0.017 |
| **Genes showing decreased expression (n=37)** | | | |
| BB0382^d^ | basic membrane protein B (*bmpB*) | -1.02 | 0.012 |
| BBS12 | hypothetical protein | -1.04 | 0.013 |
| BBO20 | conserved hypothetical protein | -1.10 | 0.011 |
| BB0328^d^ | oligopeptide ABC transporter, periplasmic oligopeptide-binding protein (*oppA-1)* | -1.16 | 0.001 |
| BB0049 | hypothetical protein | -1.24 | 0.005 |
| BBN11^d^ | hypothetical protein | -1.42 | 0.008 |
| BBM12^d^ | hypothetical protein | -1.70 | 0.02 |
| BBF20^d^ | conserved hypothetical protein | -1.98 | 0.002 |
| BB0010 | holo-[acyl-carrier protein] synthase (Holo-ACP synthase) | -2.06 | 0.016 |
| BBD21 | plasmid partition protein, putative | -2.12 | 0.002 |
| BB0366 | aminopeptidase I (*yscI*) | -2.18 | 0.013 |
| BB0536^d^ | zinc protease, putative | -2.19 | 0.006 |
| BBR41 | conserved hypothetical protein | -2.53 | 0.012 |
| BB0161 | hypothetical protein | -2.63 | 0.005 |
| BB0385 | basic membrane protein D (*bmpD*) | -2.93 | 0.007 |
| BBI11 | hypothetical protein | -2.99 | 0.019 |
| BB0364^d^ | conserved hypothetical protein | -3.14 | 0.006 |
| BBH08^d^ | hypothetical protein | -3.23 | 0.008 |
| BB0334^d^ | oligopeptide ABC transporter, ATP-binding protein (*oppD*) | -3.25 | 0.006 |
| BB0348 | pyruvate kinase (*pyk*) | -3.31 | 0.006 |
| BBI24^d^ | hypothetical protein | -3.38 | 0.008 |
| BB0139^d^ | hypothetical protein | -3.45 | 0.006 |
| BBA54^d^ | hypothetical protein | -3.45 | 0.005 |
| BBI29^d^ | hypothetical protein | -3.58 | 0.002 |
| BBK01^d^ | hypothetical protein | -3.60 | 0.002 |
| BB0226^d^ | seryl-tRNA synthetase (*serS*) | -3.69 | 0.005 |
| BB0259^d^ | hypothetical protein | -3.98 | 0.003 |
| BBI14^d^ | hypothetical protein | -4.10 | 0.001 |
| BBI39^d^ | hypothetical protein | -4.12 | 0.002 |
| BBD13^d^ | hypothetical protein | -4.13 | 0.004 |
| BBS05 | hypothetical protein | -4.37 | 0.004 |
| BBA53^d^ | hypothetical protein | -4.46 | 0.001 |
| BB0240^d^ | glycerol uptake facilitator (*glpF*) | -4.54 | <0.001 |
| BBI18^d^ | hypothetical protein | -4.62 | <0.001 |
| BBA52^d^ | outer membrane protein | -5.42 | <0.001 |
| BB0243^d^ | glycerol-3-phosphate dehydrogenase, anaerobic (*glpA*) | -6.52 | <0.001 |
| BB0241^d^ | glycerol kinase (*glpK*) | -8.27 | <0.001 |

a. Also showed increased expression in stationary phase.

b. Expression values for *blyA* orthologs (BBN23, BBR23, BBS23) were considered as a single transcript because they are 100% identical in sequence. BBR23 and BBS23 also showed increased expression in stationary phase.

c. Expression values for *blyB* orthologs (BBN24, BBP24, BBR24, BBS24) were considered as a single transcript because they are 100% identical in sequence. BBN24, BBR24, and BBS23 were also iin stationary phase.

d. Also showed decreased expression in stationary phase.
