# Supplementary material for: Characterization of the RelBbu Regulon in Borrelia burgdorferi Reveals Modulation of Glycerol Metabolism by (p)ppGpp
Source: PLoS One. 2015 Feb 17;10(2):e0118063. doi: 10.1371/journal.pone.0118063 (PMC4331090; doi:10.1371/journal.pone.0118063)
Supplement: S2 Table — (DOC) [file pone.0118063.s002.doc]

| **Table S2.** Genes modulated in *B. burgdorferi* 297 Δ*rel_Bbu_* during stationary phase of growth in vitro at 34°C by microarray analysis | | | | |
| --- | --- | --- | --- | --- |
| **ID** | **Description** | | **Mean expression** | **P** |
|  |  |  | **[log2 Δ*rel_Bbu_*/WT)]** |  |
| **Genes showing increased expression (n=174)** | | | | |
| BBB07 | | α3β1integrin-binding protein | 6.02 | <0.001 |
| BB0642 | | spermidine/putrescine ABC transporter, ATP-binding protein (*potA*) | 5.73 | <0.001 |
| BB0739 | | hypothetical protein | 5.71 | <0.001 |
| BB0807 | | conserved hypothetical integral membrane protein | 5.42 | 0.015 |
| BBP03 | | hypothetical protein | 5.26 | <0.001 |
| BBO42 | | hypothetical protein | 5.20 | 0.015 |
| BBA09 | | conserved hypothetical protein | 5.12 | 0.002 |
| BBB05^a^ | | chitibiose transporter protein *chbA* | 5.07 | <0.001 |
| BBB06 | | chitibiose transporter protein *chbB* | 5.02 | <0.001 |
| BB0579 | | DNA polymerase III, α subunit (*dnaE*) | 4.95 | 0.001 |
| BBP25 | | conserved hypothetical protein | 4.83 | 0.012 |
| BB0580 | | conserved hypothetical integral membrane protein | 4.79 | 0.01 |
| BBP02 | | hypothetical protein | 4.77 | 0.01 |
| BBP40 | | hypothetical protein | 4.63 | 0.001 |
| BB0532 | | hypothetical protein | 4.46 | 0.001 |
| BB0806 | | hypothetical protein | 4.46 | <0.001 |
| BBO38 | | conserved hypothetical protein | 4.41 | 0.016 |
| BB0685 | | 3-hydroxy-3-methylglutaryl-CoA reductase (*mvaA*) | 4.39 | <0.001 |
| BBP30 | | conserved hypothetical protein | 4.38 | 0.001 |
| BBR02 | | hypothetical protein, authentic frameshift | 4.36 | 0.001 |
| BB0640^a^ | | spermidine/putrescine ABC transporter, permease protein (*potC*) | 4.34 | <0.001 |
| BBS01 | | hypothetical protein | 4.22 | <0.001 |
| BB0262 | | conserved hypothetical protein | 4.20 | <0.001 |
| BB0467 | | conserved hypothetical protein | 4.09 | 0.014 |
| BBR43 | | hypothetical protein | 4.07 | 0.015 |
| BBS03 | | hypothetical protein | 4.03 | 0.01 |
| BB0158 | | antigen S2, putative | 4.00 | 0.003 |
| BBP04 | | hypothetical protein | 4.00 | 0.002 |
| BBO03 | | hypothetical protein | 3.91 | <0.001 |
| BB0297 | | small GTP-binding protein (*smg*) | 3.86 | <0.001 |
| BB0427 | | conserved hypothetical protein | 3.82 | <0.001 |
| BB0808 | | hypothetical protein | 3.82 | 0.014 |
| BBN01 | | hypothetical protein | 3.82 | 0.001 |
| BB0177 | | glucose inhibited division protein B (*gidB*) | 3.81 | 0.007 |
| BB0776^a^ | | hypothetical protein | 3.70 | <0.001 |
| BBP01 | | hypothetical protein | 3.63 | 0.003 |
| BBA11 | | conserved hypothetical protein | 3.59 | <0.001 |
| BB0777 | | adenine phosphoribosyltransferase (*apt*) | 3.58 | <0.001 |
| BBM01 | | hypothetical protein | 3.57 | <0.001 |
| BB0406 | | hypothetical protein | 3.54 | <0.001 |
| BBO01 | | hypothetical protein | 3.45 | 0.002 |
| BB0251 | | leucyl-tRNA synthetase (*leuS*) | 3.41 | <0.001 |
| BB0429^a^ | | hypothetical protein | 3.38 | <0.001 |
| BBO21 | | conserved hypothetical protein | 3.38 | <0.001 |
| BB0459 | | hypothetical protein | 3.33 | 0.001 |
| BB0747 | | oligopeptide ABC transporter, permease protein (*oppB-2*) | 3.32 | <0.001 |
| BB0168^a^ | | *dnaK* suppressor (*dksA*) | 3.26 | <0.001 |
| BBM02 | | hypothetical protein | 3.24 | <0.001 |
| BB0554 | | hypothetical protein | 3.21 | 0.001 |
| BB0205 | | hypothetical protein | 3.16 | <0.001 |
| BB0163 | | hypothetical protein | 3.15 | 0.019 |
| BB0748 | | hypothetical protein | 3.14 | 0.002 |
| BBS06 | | conserved hypothetical protein | 3.12 | 0.001 |
| BB0664 | | hypothetical protein | 3.10 | <0.001 |
| BB0159 | | antigen S2-related protein | 3.09 | 0.004 |
| BBO04 | | hypothetical protein | 3.08 | 0.003 |
| BB0178 | | glucose inhibited division protein A (*gidA*) | 3.07 | 0.005 |
| BBB12 | | plasmid partition protein, putative | 3.07 | 0.001 |
| BBN29 | | hypothetical protein, authentic point mutation | 3.07 | 0.008 |
| BB0578 | | methyl-accepting chemotaxis protein (*mcp-1*) | 3.06 | 0.012 |
| BB0830 | | exonuclease SbcC (*sbcC*) | 3.04 | 0.008 |
| BBM08 | | conserved hypothetical protein | 3.04 | 0.001 |
| BB0179 | | thiophene and furan oxidation protein (*thdF*) | 3.03 | <0.001 |
| BB0782 | | conserved hypothetical protein | 2.99 | <0.001 |
| BBB23 | | conserved hypothetical protein | 2.99 | 0.013 |
| BBA13 | | conserved hypothetical protein | 2.98 | <0.001 |
| BB0302 | | cell division protein (*ftsW*) | 2.96 | 0.001 |
| BB0722 | | hypothetical protein | 2.95 | 0.017 |
| BB0783 | | hypothetical protein | 2.93 | 0.002 |
| BBB18 | | GMP synthase (*guaA*) | 2.92 | <0.001 |
| BBM21 | | conserved hypothetical protein | 2.92 | 0.003 |
| BBA14 | | conserved hypothetical protein | 2.88 | <0.001 |
| BB0066 | | hypothetical protein | 2.87 | <0.001 |
| BB0615 | | ribosomal protein S4 (*rpsD*) | 2.87 | <0.001 |
| BB0433 | | hypothetical protein | 2.85 | 0.011 |
| BBR29 | | conserved hypothetical protein | 2.83 | 0.003 |
| BBA12 | | conserved hypothetical protein | 2.81 | 0.004 |
| BB0344 | | DNA helicase (*uvrD*) | 2.79 | 0.006 |
| BBP15 | | hypothetical protein | 2.75 | 0.005 |
| BB0063 | | hypothetical protein | 2.66 | <0.001 |
| BB0839 | | hypothetical protein | 2.66 | 0.003 |
| BB0118 | | zinc protease, putative | 2.64 | 0.001 |
| BB0552 | | DNA ligase (*lig*) | 2.64 | 0.01 |
| BB0424 | | hypothetical protein | 2.62 | 0.003 |
| BB0022 | | Holliday junction DNA helicase (*ruvB*) | 2.58 | <0.001 |
| BBP32 | | plasmid partition protein, putative | 2.55 | 0.001 |
| BBO29 | | hypothetical protein | 2.54 | 0.019 |
| BB0726^a^ | | *minD*-related ATP-binding protein (*ylxH-3*) | 2.52 | 0.001 |
| BB0137 | | long-chain-fatty-acid CoA ligase | 2.51 | 0.013 |
| BB0784 | | conserved hypothetical protein | 2.51 | 0.002 |
| BBO06 | | conserved hypothetical protein | 2.51 | <0.001 |
| BB0084 | | Nitrogen fixation regulon protein (*nifS*) | 2.48 | 0.017 |
| BB0304 | | UDP-N-acetylmuramoylalanyl-D-glutamyl-2,6-diaminopimelate-D-alanyl-D-alanine ligase (*murF*) | 2.48 | 0.001 |
| BB0555 | | hypothetical protein | 2.48 | 0.012 |
| BB0525^a^ | | conserved hypothetical protein | 2.47 | 0.001 |
| BB0420^a^ | | sensory transduction histidine kinase/response regulator (­*hk1*) | 2.39 | <0.001 |
| BB0836 | | excinuclease ABC, B subunit (*uvrB*) | 2.35 | <0.001 |
| BB0029 | | conserved hypothetical protein | 2.34 | 0.019 |
| BB0217 | | phosphate ABC transporter, permease protein (*pstA*) | 2.34 | <0.001 |
| BB0684 | | carotenoid biosynthesis protein, putative | 2.32 | <0.001 |
| BB0421 | | hydrolase | 2.20 | 0.003 |
| BB0712 | | RNA polymerase σ^70^ factor (*rpoD*) | 2.19 | <0.001 |
| BBP21 | | conserved hypothetical protein | 2.14 | 0.019 |
| BB0340 | | hypothetical protein | 2.13 | <0.001 |
| BBB04^a^ | | chitobiose transporter protein *chbC* | 2.11 | <0.001 |
| BB0336 | | antigen P26 | 2.08 | 0.002 |
| BB0744 | | antigen p83/100 | 2.06 | 0.007 |
| BB0518 | | heat shock protein 70 (*dnaK*-2) | 2.05 | 0.001 |
| BB0670 | | purine-binding chemotaxis protein (*cheW*-3) | 2.03 | 0.011 |
| BB0343^a^ | | Glu-tRNA(Gln) amidotransferase, subunit C (*gatC*) | 2.02 | <0.001 |
| BB0790 | | hypothetical protein | 1.99 | <0.001 |
| BBO08 | | conserved hypothetical protein | 1.97 | <0.001 |
| BBP20 | | conserved hypothetical protein | 1.97 | <0.001 |
| BBO02^a^ | | hypothetical protein | 1.96 | 0.002 |
| BB0271 | | flagellar biosynthesis protein (*flhA*) | 1.95 | 0.002 |
| BB0781^a^ | | GTP-binding protein (*obg*) | 1.95 | <0.001 |
| BBB02 | | hypothetical chitobiose | 1.91 | 0.007 |
| BB0727 | | pyrophosphate--fructose 6-phosphate 1-phosphotransferase (*pfk*) | 1.88 | 0.005 |
| BBS20 | | conserved hypothetical protein | 1.84 | 0.006 |
| BBR20 | | conserved hypothetical protein | 1.83 | 0.002 |
| BB0059 | | hemolysin (*tlyC*) | 1.77 | <0.001 |
| BBM20 | | conserved hypothetical protein | 1.75 | <0.001 |
| BB0055 | | triosephosphate isomerase | 1.72 | 0.011 |
| BB0296 | | heat shock protein (*hslV*) | 1.72 | <0.001 |
| BB0639 | | spermidine/putrescine ABC transporter, spermidine/putrescine-binding periplasmic protein (*potD*) | 1.72 | <0.001 |
| BBR19 | | conserved hypothetical protein | 1.72 | 0.015 |
| BB0821 | | 2-methylthio-N6-isopentyladenosine tRNA modification enzyme (*miaA*) | 1.70 | 0.01 |
| BB0683^a^ | | 3-hydroxy-3-methylglutaryl-CoA synthase (*hmgs*) | 1.65 | <0.001 |
| BB0514 | | phenylalanyl-tRNA synthetase, β subunit (*pheT*) | 1.63 | 0.009 |
| BB0795 | | outer membrane protein | 1.62 | <0.001 |
| BBA23^a^ | | conserved hypothetical protein | 1.61 | <0.001 |
| BBM19 | | conserved hypothetical protein | 1.61 | <0.001 |
| BB0711^a^ | | hypothetical protein | 1.60 | <0.001 |
| BBB17 | | IMP dehydrogenase (*guaB*) | 1.60 | <0.001 |
| BB0840 | | hypothetical protein | 1.59 | 0.004 |
| BB0438^a^ | | DNA polymerase III, β subunit (*dnaN*) | 1.56 | <0.001 |
| BB0229 | | ribosomal protein L31 (*rpmE*) | 1.54 | <0.001 |
| BB0542 | | hypothetical protein | 1.52 | 0 |
| BBN21 | | hypothetical protein, authentic frameshift | 1.52 | <0.001 |
| BB0778 | | ribosomal protein L21 (*rplU*) | 1.51 | 0.002 |
| BB0130 | | hypothetical protein | 1.50 | <0.001 |
| BBS21^a^ | | conserved hypothetical protein | 1.46 | <0.001 |
| BB0724 | | K+ transport protein (*ntpJ*) | 1.43 | <0.001 |
| BBM23^b^ | | pore-forming hemolysin (*blyA*) | 1.43 | <0.001 |
| BB0419 | | response regulatory protein (*rrp-1*) | 1.42 | 0.001 |
| BBP31 | | conserved hypothetical protein | 1.42 | 0.006 |
| BB0216 | | phosphate ABC transporter, permease protein (*pstC*) | 1.40 | 0.003 |
| BB0093 | | V-type ATPase, subunit B (*atpB*) | 1.39 | 0.016 |
| BB0067 | | peptidase, putative | 1.38 | 0.001 |
| BBN20^a^ | | conserved hypothetical protein | 1.38 | 0.005 |
| BBP19 | | conserved hypothetical protein | 1.37 | 0.004 |
| BB0442 | | inner membrane protein | 1.36 | <0.001 |
| BB0824 | | hypothetical protein | 1.34 | 0.01 |
| BB0710^a^ | | DNA primase (*dnaG*), authentic frameshift | 1.33 | <0.001 |
| BB0133 | | hypothetical protein | 1.32 | <0.001 |
| BBA39 | | hypothetical protein | 1.26 | 0.008 |
| BB0466 | | ABC transporter, ATP-binding protein | 1.24 | 0.001 |
| BB0713 | | conserved hypothetical protein | 1.22 | 0.002 |
| BB0342 | | Glu-tRNA(Gln) amidotransferase, subunit A (*gatA*) | 1.20 | 0.016 |
| BB0441^a^ | | ribonuclease P protein component (*rnpA*) | 1.20 | <0.001 |
| BB0027 | | hypothetical protein | 1.17 | <0.001 |
| BB0788 | | conserved hypothetical protein | 1.17 | <0.001 |
| BBN24^a,c^ ^b^ | | hemolysin accessory protein (*blyB*) | 1.17 | <0.001 |
| BB0065 | | polypeptide deformylase (*def*) | 1.14 | <0.001 |
| BBP34 | | conserved hypothetical protein | 1.13 | 0.013 |
| BB0779 | | hypothetical protein | 1.04 | 0.006 |
| BB0837 | | excinuclease ABC, A subunit (*uvrA*) | 1.04 | 0.003 |
| BB0775 | | flagellar hook-basal body complex protein (*flhO*) | 1.03 | <0.001 |
| BB0780 | | ribosomal protein L27 (*rpmA*) | 1.03 | <0.001 |
| BB0042 | | phosphate transport system regulatory protein (*phoU*) | 1.02 | 0.003 |
| BB0044 | | hypothetical protein | 1.02 | <0.001 |
| BB0088 | | GTP-binding membrane protein (*lepA*) | 1.01 | <0.001 |
| BB0789 | | cell division protein (*ftsH*) | 1.01 | <0.001 |
| BB0691 | | translation elongation factor G (*fus-2*) | 0.96 | 0.001 |
| **Genes showing decreased expression (n=103)** | | | | |
| BB0705 | | ribonuclease III (*rnc*) | -0.96 | <0.001 |
| BB0359 | | carboxyl-terminal protease (*ctp*) | -0.97 | <0.001 |
| BB0407 | | mannose-6-phosphate isomerase (*manA*) | -0.98 | <0.001 |
| BBM12^d^ | | hypothetical protein | -0.98 | 0.006 |
| BB0463 | | nucleoside-diphosphate kinase (*ndk*) | -1.00 | <0.001 |
| BB0473 | | conserved hypothetical integral membrane protein | -1.03 | 0.003 |
| BB0462 | | conserved hypothetical protein | -1.05 | 0.009 |
| BB0346 | | hypothetical protein | -1.07 | 0.002 |
| BBS11 | | hypothetical protein | -1.09 | 0.004 |
| BB0508 | | GTP-binding protein | -1.10 | 0.014 |
| BBP11 | | hypothetical protein | -1.11 | 0.008 |
| BB0194 | | conserved hypothetical protein | -1.13 | 0.018 |
| BB0668 | | flagellar filament outer layer protein (*flaA*) | -1.13 | <0.001 |
| BB0181 | | flagellar hook-associated protein (*flgK*) | -1.14 | 0.01 |
| BB0383 | | basic membrane protein A (*bmpA*) | -1.15 | 0.016 |
| BBO11 | | hypothetical protein | -1.15 | 0.006 |
| BB0461 | | DNA polymerase III, γ and τ subunits (*dnaX*) | -1.17 | 0.001 |
| BBM14 | | hypothetical protein | -1.17 | 0.016 |
| BB0020 | | pyrophosphate--fructose 6-phosphate 1-phosphotransferase, β subunit (*pfpB*) | -1.18 | <0.001 |
| BB0195 | | conserved hypothetical protein | -1.19 | <0.001 |
| BB0756 | | hypothetical protein | -1.19 | 0.007 |
| BB0334^d^ | | oligopeptide ABC transporter, ATP-binding protein (*oppD*) | -1.21 | 0.002 |
| BB0337 | | enolase (*eno*) | -1.24 | 0.002 |
| BBO34 | | conserved hypothetical protein | -1.25 | 0.005 |
| BB0259^d^ | | hypothetical protein | -1.29 | <0.001 |
| BB0147 | | flagellar filament 41 kDa core protein (*flaB*) | -1.31 | 0.001 |
| BB0329 | | oligopeptide ABC transporter, periplasmic oligopeptide-binding protein (*oppA*-2) | -1.33 | <0.001 |
| BBN11^d^ | | hypothetical protein | -1.33 | 0.017 |
| BBD12 | | hypothetical protein | -1.34 | 0.007 |
| BB0448 | | phosphocarrier protein HPr (*ptsH*-1) | -1.35 | 0.001 |
| BB0006 | | conserved hypothetical integral membrane protein | -1.37 | <0.001 |
| BB0197 | | protoporphyrinogen oxidase, putative | -1.46 | <0.001 |
| BBK13 | | conserved hypothetical protein | -1.47 | 0.001 |
| BB0335 | | oligopeptide ABC transporter, ATP-binding protein (*oppF*) | -1.51 | <0.001 |
| BB0203 | | Lambda CII stability-governing protein (*hflK*) | -1.53 | 0.02 |
| BBA16 | | outer surface protein B (*ospB*) | -1.54 | 0.002 |
| BB0364^d^ | | conserved hypothetical protein | -1.57 | <0.001 |
| BBA61 | | conserved hypothetical protein | -1.57 | 0.001 |
| BB0382^d^ | | basic membrane protein B (*bmpB*) | -1.59 | <0.001 |
| BBA18 | | conserved hypothetical protein | -1.63 | <0.001 |
| BB0139^d^ | | hypothetical protein | -1.74 | <0.001 |
| BBA15 | | outer surface protein A (*ospA*) | -1.81 | 0.001 |
| BB0226^d^ | | seryl-tRNA synthetase (*serS*) | -1.82 | 0.001 |
| BB0452 | | hypothetical protein | -1.83 | <0.001 |
| BBK15 | | antigen P35, putative | -1.83 | <0.001 |
| BBF20^d^ | | conserved hypothetical protein | -1.84 | 0.001 |
| BBK19 | | hypothetical protein | -1.86 | 0.001 |
| BBD18 | | hypothetical protein | -1.87 | 0.002 |
| BBA68 | | hypothetical protein | -1.88 | 0.002 |
| BBD13^d^ | | hypothetical protein | -1.92 | 0.002 |
| BBO27 | | conserved hypothetical protein | -1.97 | 0.016 |
| BB0367 | | hypothetical protein | -1.99 | 0.01 |
| BB0365 | | lipoprotein LA7 | -2.02 | <0.001 |
| BBA19 | | conserved hypothetical protein | -2.02 | <0.001 |
| BBA74 | | membrane-associated periplasmic protein | -2.03 | <0.001 |
| BB0631 | | hypothetical protein | -2.04 | <0.001 |
| BBA20 | | plasmid partition protein, putative | -2.09 | 0.001 |
| BBI24^d^ | | hypothetical protein | -2.13 | <0.001 |
| BB0760 | | hypothetical protein | -2.14 | <0.001 |
| BB0536^d^ | | zinc protease, putative | -2.21 | <0.001 |
| BB0007 | | hypothetical protein | -2.27 | 0.001 |
| BB0024 | | hypothetical protein | -2.30 | <0.001 |
| BB0409 | | hypothetical protein | -2.43 | 0.004 |
| BBA59 | | lipoprotein | -2.56 | <0.001 |
| BBA62 | | lipoprotein | -2.58 | <0.001 |
| BB0328^d^ | | oligopeptide ABC transporter, periplasmic oligopeptide-binding protein (*oppA*-1) | -2.65 | 0.002 |
| BBR18 | | hypothetical protein | -2.65 | 0.014 |
| BBK18 | | conserved hypothetical protein | -2.80 | <0.001 |
| BBD11 | | conserved hypothetical protein | -2.86 | 0.001 |
| BBA03 | | outer membrane protein | -2.97 | <0.001 |
| BBJ18 | | conserved hypothetical protein | -3.00 | 0.003 |
| BBP28 | | lipoprotein | -3.07 | <0.001 |
| BBA53^d^ | | hypothetical protein | -3.11 | 0.002 |
| BBO33 | | conserved hypothetical protein | -3.22 | 0.012 |
| BBA54^d^ | | hypothetical protein | -3.30 | 0.008 |
| BBA52^d^ | | outer membrane protein | -3.31 | 0.011 |
| BBA69 | | hypothetical protein | -3.55 | <0.001 |
| BB0243^d^ | | glycerol-3-phosphate dehydrogenase (*glpD*) | -3.79 | <0.001 |
| BBR10 | | conserved hypothetical protein | -3.97 | 0.003 |
| BB0330 | | oligopeptide ABC transporter, periplasmic oligopeptide-binding protein (*oppA*-3) | -4.07 | 0.015 |
| BBK36 | | hypothetical protein | -4.18 | 0.017 |
| BBE16 | | hypothetical protein | -4.39 | <0.001 |
| BBE17 | | hypothetical protein | -4.55 | 0.005 |
| BB0240^d^ | | glycerol uptake facilitator (*glpF*) | -4.76 | <0.001 |
| BBA60 | | surface lipoprotein P27 | -5.07 | <0.001 |
| BBH18 | | hypothetical protein | -5.2 | 0.01 |
| BBH29 | | conserved hypothetical protein | -5.29 | 0.006 |
| BB0241^d^ | | glycerol kinase (*glpK*) | -5.47 | <0.001 |
| BBI23 | | hypothetical protein | -6.11 | 0.003 |
| BBH13 | | conserved hypothetical protein | -7.2 | <0.001 |
| BBH08^d^ | | hypothetical protein | -7.35 | 0.001 |
| BBH06 | | hypothetical protein | -8 | 0.001 |
| BBI38 | | hypothetical protein | -8.11 | 0.001 |
| BBI14^d^ | | hypothetical protein | -8.53 | <0.001 |
| BBJ41 | | antigen P35, putative | -8.62 | <0.001 |
| BBH26 | | hypothetical protein | -8.82 | 0.008 |
| BBK01^d^ | | hypothetical protein | -8.85 | <0.001 |
| BBI18^d^ | | hypothetical protein | -9.08 | 0.001 |
| BBI39^d^ | | hypothetical protein | -9.1 | <0.001 |
| BBH37 | | hypothetical protein | -9.12 | 0.001 |
| BBH28 | | plasmid partition protein, putative | -9.13 | <0.001 |
| BBI36 | | antigen P35, putative | -9.64 | <0.001 |
| BBI29^d^ | | hypothetical protein | -10.51 | 0.001 |

a. Also showed increased expression in exponential phase.

b. Expression values for *blyA* orthologs (BBM23, BBP23, BBR23) were considered as a single transcript because they are 100% identical in sequence. BBR23 and BBS23 also showed increased expression in exponential phase.

c. Expression values for *blyB* orthologs (BBN24, BBR24, BBS24) were considered as a single transcript because they are 100% identical in sequence. BBN24, BBR24, and BBS23 also showed increased expression in exponential phase.

d. Also showed increased expression in exponential phase.
