# Supplementary material for: Characterization of the RelBbu Regulon in Borrelia burgdorferi Reveals Modulation of Glycerol Metabolism by (p)ppGpp
Source: PLoS One. 2015 Feb 17;10(2):e0118063. doi: 10.1371/journal.pone.0118063 (PMC4331090; doi:10.1371/journal.pone.0118063)
Supplement: S3 Table — (DOC) [file pone.0118063.s003.doc]

| **Table S3**. Primers used in this study | | | |
| --- | --- | --- | --- |
| Primer | Sequence (5'-3') | Gene | Gene location |
| BB0147qF | GCAGCTAATGTTGCAAATCTTTTC | *flaB* (BB0147, flagellar antigen) | Chromosome |
| BB0147qR | GCAGGTGCTGGCTGTTGA |  |
| BB0168F | GCAAAAAGCTGTTTCTGAGCATGAG | *dksA* (BB0168, *dnaK* suppressor) | Chromosome |
| BB0168R | GGAATAGCTAAAAGTCTCTCCCTAGC |  |
| BB198qF2 | ATTTTCACACTCAATGACGTAAGAC | *rel* (BB0198, (p)ppGpp synthetase/hydrolase) | Chromosome |
| BB198qR2 | AAGATATTTGGGTTTTCTTTTAAAGCAG |  |
| BB198qF3 | ATTGCCTTTCAACTTACGTTCC |  |
| BB198qR3 | AGATGCTTAAATGAAAGATCCTCAAG |  |
| BB0240qF | AAGTCCCGAAATACCAGGAG | *glpF* (BB0240, glycerol facilitator) | Chromosome |
| BB0240qR | TTCTTGCTGCTGTGTAAATACC |  |  |
| BB0241F | TTATCTATTGATCAAGGTACTACTAGCTCG | *glpK* (BB0241, glycerol kinase) | Chromosome |
| BB0241R | CCTGTATTTTTTTCCCATATAACCG |  |
| BB0243F | CAACAGGTCTTGGCATTGCGGTAGA | *glpD* (BB0243, glycerol-3-phosphate dehydrogenase, aerobic) | Chromosome |
| BB0243R | GGCCCTCTGTTTTAATATTGGGAGC |  |
| BB0243qF | GCTCTGTTCTATATTACGATGATT |  |
| BB0243qR | AGGGCAATGCCTCCTTTTT |  |
| BB0330F | ATTCTCAGGGCTTTTGACATTAAATACC | *oppA-*3 (BB0330, oligopeptide ABC transporter) | Chromosome |
| BB0330R | CAGCGTTTTACTATCAATTGCCTTG |  |
| BB0335F | TAGGACTCGTAGGAGAATCTGGTTG | *oppF* (BB0335, oligopeptide ABC transporter) | Chromosome |
| BB0335R | TTCCTATTCTTTGTCTTTGTCCTCC |  |
| BB0385F | AAACTAAACCAAGACCATCTTCTTTAAGCC | *bmpD* (BB0385, basic membrane protein D) | Chromosome |
| BB0385R | GGGTTGATATCATATTTGCAGCTGC |  |
| BB0640F | TGCAGAAATAGCATTTATTACGGGTTTG | *potC* (BB0640, spermidine/putrescine ABC transporter) | Chromosome |
| BB0640R | CCTGACATTGTAACAGGAATAAGCTTAA |  |
| BB0642F | TCCTTTTGGAAGTAGTTTTACATCTTCTGG | *potA* (BB0642, spermidine/putrescine ABC transporter) | Chromosome |
| BB0642R | TTGACAATGAGTGACAGAATCGTTG |  |
| BB0685F | GCCAATTGCAACAGAAGAATCTTC | *mvaA* (BB0685, 3-hydroxy-3-methylglutaryl-CoA reductase) | Chromosome |
| BB0685R | CACGCTCTGCAATTGAGTTTAGC |  |
| BB0712F | TTAACGGGAAATCAAGAAGTTGAAC | *rpoD* (BB0712, RNA polymerase σ^70^ factor) | Chromosome |
| BB0712R | CCTCTTGATACAAAGGAATGTCTCTC |  |
| BB0777F | AGATCGATTGCATTGCAGTTGTTG | *apt* (BB0777, adenine phosphoribosyltransferase) | Chromosome |
| BB0777R | GGGAATTGACTTCATAACTTTCAAGAC |  |
| BBA03F | ATCAAATCCGCTAGCAAGTATGC | BBA03, outer membrane protein | lp54 |
| BBA03R | CAAAAACAAAAGTAATGATGCCGAGC |  |
| BBA74F | ATGTGTTTGCAGATTCTAACAATGC | BBA74, periplasmic protein | lp54 |
| BBA74R | AACAGTCCCTTTAGCAACATCTGAC |  |
| BBB04F | TATAGGATTTAAAACTATTGGTGCACCG | *chbC* (BBB04, PTS system, chitobiose-specific IIC component) | cp26 |
| BBB04R | ATGGTTTTGTGGTCTTCATGGTACC |  |
| BBB07F | CTTGGCGCCTGGGCAATTAGATTAG | BBB07, outer surface protein, putative | cp26 |
| BBB07R | CTAATAAATGCGGCTGTTGGGATTG |  |
| BBM23F | ATGGATACTATTAAATTAACCGAACTTC | BBM23, pore-forming hemolysin (*blyA*) | cp32-6 |
| BBM23R | CCATTACCATTCTTAAAAATCTTGCC |  |
